# Induction of sestrin 2 is associated with fisetin-mediated apoptosis in human head and neck cancer cell lines

**DOI:** 10.3164/jcbn.18-63

**Published:** 2018-10-13

**Authors:** Dong-Hoon Won, Shin Hye Chung, Ji-Ae Shin, Kyoung-Ok Hong, In-Hyoung Yang, Jun-Won Yun, Sung-Dae Cho

**Affiliations:** 1 Department of Biotechnology, Catholic University of Korea, 43 Jibong-ro, Bucheon-si, Gyeonggi-do 14662, Republic of Korea; 2 Department of Dental Biomaterials Science, School of Dentistry and Dental Research Institute, Seoul National University, 103 Daehak-ro, Jongno-gu, Seoul 03080, Republic of Korea; 3 Department of Oral Pathology, School of Dentistry and Dental Research Institute, Seoul National University, 103 Daehak-ro, Jongno-gu, Seoul 03080, Republic of Korea; 4 Department of Oral Pathology, School of Dentistry, Institute of Oral Bioscience, Chonbuk National University, Jeonju 54896, Republic of Korea

**Keywords:** fisetin, sestrin 2, human head and neck cancer, apoptosis

## Abstract

Fisetin was reported to have an anti-proliferative and apoptotic activity as a novel anti-cancer agent in various cancer cell lines. However, the possible molecular targets for the anti-cancer effect of fisetin in human head and neck cancer (HNCC) have not yet been clarified. In this study, the influence of fisetin on the growth and apoptosis of HNCCs were examined. In HSC3 cells, fisetin treatment reduced the viability and induced apoptosis. Through the results from the screening of the expression profile of apoptosis-related genes, sestrin 2 (SESN2) was functionally involved in fisetin-mediated apoptosis showing the knockdown of SESN2 by siRNA clearly restored fisetin-induced apoptosis. In addition, fisetin reduced the protein expression levels of phospho-mTOR (p-mTOR) and Mcl-1, which are the downstream molecules of SESN2. It also induced PARP cleavage by inducing an increase in the expression levels of SESN2 together with reducing mTOR and Mcl-1 proteins in other three HNCCs (MC3, Ca9.22, and HN22). Taken together, our findings suggest that the anti-cancer effect of fisetin on HNCCs is associated with SESN2/mTOR/Mcl-1 signaling axis.

## Introduction

Sestrins (SESNs), a protein family composed of SESN1, SESN2, and SESN3 in mammals, are conserved stress-responsive proteins that reduce reactive oxygen species (ROS) and are involved in the regulation of cell survival.^(1)^ SESN2, a homolog of p53-activated gene 26, is induced by cytotoxic events such as hypoxia, DNA damage, and oxidative stress.^(2,3)^ SESN2 is down-regulated in cancer cells as a result of the increased production and accumulation of ROS and the redox state of tumor cells.^(4,5)^ Similarly, a lack of SESN2 in mouse embryonic fibroblasts increases proliferation of RAS-activated tumor cells.^(6)^ Recently, several studies have reported that SESN2 suppressed cell proliferation and was involved in apoptosis in colon cancer cells.^(1,7)^ However, the relationship between SESN2 and oral cancer is not clear.

Fisetin (3,3',4',7-tetrahydroxyflavone) is a naturally occurring flavonoid found in numerous vegetables and fruits such as apple, onion, grape, cucumber, persimmon, and strawberry.^(8,9)^ Anti-diabetic, cardio-protective, and neuro-protective activities of fisetin have been demonstrated by using *in vitro* and *in vivo* experimental models relevant to human diseases.^(10–12)^ A potential against cell growth and survival of various cancer cells has been shown.^(13–15)^ Recently, fisetin inhibited malignant proliferation in human oral squamous cell carcinoma cell lines through inhibition of Met/Src signaling pathways.^(16)^ However, crucial molecular targets for the anticancer effect of fisetin have not been identified on human head and neck cancer cells (HNCCs).

Here, the anticancer activity and the molecular targets of fisetin in HNCCs were investigated *in vitro*. Our results indicate that fisetin induces apoptosis in HNCCs by upregulating SESN2.

## Materials and Methods

### Cell culture and chemical treatment

**Fig. 1:**
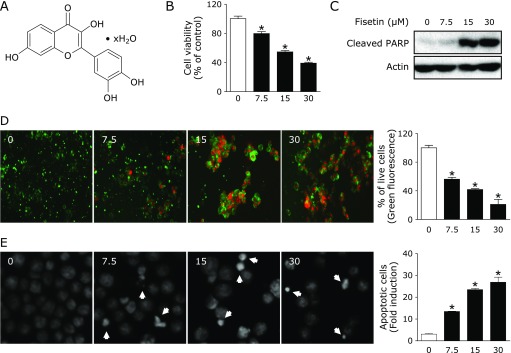
A role of fisetin in growth inhibition and apoptotic induction on HSC3 cells. (A) The chemical structure of fisetin. The empirical formula of fisetin is C_15_H_10_O_6_ · xH_2_O. (B) Cell viability was examined using the trypan blue exclusion assay. (C) The protein expression of cleaved PARP was detected using western blotting and β-actin was used as a loading control. (D) Cytotoxicity of fisetin was measured using a Live/Dead assay. Fluorescence images of HSC3 cells were observed under a fluorescence microscopy (Magnification, ×200). (E) Apoptotic cells stained with DAPI solution were observed by a fluorescence microscope (Magnification, ×400). The all graphs were expressed as the means of three independent experiments. *p<0.05.

HSC3 and Ca9.22 Human oral squamous cell carcinoma cell lines were provided from Prof. Shindoh (Hokkaido University, Sapporo, Japan). MC3 mucoepidermoid carcinoma cell line was provided by prof. Wu Junzheng (Forth Military Medical University, Xi’an, China), and HN22 human head and neck squamous cell carcinoma cell line was obtained from Prof. Lee (Dankook University, Cheonan, Korea). The cells were cultured in DMEM supplemented with 10% fetal bovine serum (FBS) and antibiotics at 37°C in 5% CO_2_ incubator. All experiments were prepared after the cells reached 50~60% confluence. Fisetin (Fig. 1A; Sigma-Aldrich, St. Louis, MO) was dissolved in dimethyl sulfoxide (DMSO), aliquoted, and stored at −20°C. Final concentration of DMSO did not exceed 0.1%.

### Trypan blue exclusion assay

The growth inhibitory effects of fisetin were determined with trypan blue solution (Gibco, Paisley, UK). Cells were stained with trypan blue (0.4%), and viable cells were counted using a hemocytometer.

### Western blotting

Whole-cell lysates were prepared with a lysis buffer and protein concentration of each sample was measured using a *DC* Protein Assay Kit (BIO-RAD Laboratories, Madison, WI). After normalization, equal amount of protein was separated by SDS-PAGE and transferred to Immuno-Blot PVDF membranes. The membranes were blocked with 5% skim milk in TBST at RT for 2 h and incubated with primary antibodies and corresponding HRP-conjugated secondary antibodies. Antibodies against cleaved PARP, cleaved caspase-3, SESN2, p-mTOR, mTOR, and Mcl-1 were purchased from Cell Signaling Technology, Inc. (Charlottesville, VA) and actin antibody was obtained from Santa Cruz Biotechnology, Inc. (Santa Cruz, CA). The immunoreactive bands were visualized by ImageQuant LAS 500 (GE Healthcare Life Sciences, Piscataway, NJ).

### Live/dead assay

The cytotoxicity of fisetin was determined using a Live/Dead Viability/Cytotoxicity assay kit (Life Technologies, Grand Island, NY). The polyanionic dye, calcein-AM is retained within live cells, producing an intense green fluorescence through intracellular esterase activity. Ethidium homodimer-1 enters dead cells with damaged cell membranes and binds to nucleic acids, producing a bright red fluorescence. Briefly, cells were stained with 2 µM calcein-AM and 4 µM ethidium homodimer-1 and incubated for 30 min at RT. Cells were analyzed under a fluorescence microscopy (Leica DMi8, Wetzlar, Germany) with the appropriate excitation and emission filters. A total of three random photo were selected from each three independent experiments for quantification. The percentage of live cells was manually calculated by measuring the number of green fluorescence-labeled cells.

### 4'-6-Diamidino-2-phenylindole staining

To identify the changes in nuclear morphologies of apoptotic cells, the cells were stained with 4'-6-Diamidino-2-phenylindole (DAPI) solution (Sigma-Aldrich, Louis, MO). Briefly, cells were fixed with 100% methanol at RT for 10 min, deposited on slide glasses, and stained with DAPI solution (2 µg/ml). The morphological changes of apoptotic cells were observed under a fluorescence microscopy (Leica DMi8, Wetzlar, Germany).

### Microarray

Total RNA was extracted from cells using RNeasy Mini kit (Qiagen, CA) according to the manufacturer’s instructions. Two sets of samples were independently prepared and analyzed. The integrity and quantity of total RNA were assessed by Agilent 2100 Bioanalyzer and Nanodrop 1000 analyzer. For each sample, total RNA was analyzed using a Human Gene 2.0 ST Array. The GeneChip Arrays were immediately scanned with Affymetrix GeneChip Scanner 3000 7 G.

### Real-Time PCR

Total RNA was extracted using easy-BLUE Total RNA Extraction Kit (INTRON, Daejeon, Korea). The isolated RNA was transcribed by AMPIGENE cDNA Synthesis Kit (Enzo Life sciences, Inc., NY) and real time PCR was performed using the StepOne Real-Time PCR System with AMPIGENE qPCR Green Mix Hi-Rox (Enzo Life sciences, Inc., Farmingdale, NY). Real-time PCR conditions for all genes were as follows: 95°C for 2 min, followed by 40 cycles of 95°C for 10 s and 62°C for 30 s. The relative expression changes of the target genes were quantified by normalizing their expression to that of GAPDH. The PCR primers of all the target genes are listed in Table 1.

### siRNA transfection

siSESN2 and siControl were purchased from Bioneer (Daejeon, Korea). Briefly, HSC3 cells were transfected with 50 nM siRNA using Lipofectamine 3000 (Invitrogen, Carlsbad, CA) for 6 h followed by treatment with fisetin for 24 h. After the transfection, HSC3 cells were subjected to Western blotting.

### Statistical analysis

ANOVA followed by Tukey’s post hoc test were used to determine the significance between the control and treatment groups; *p* value of <0.05 was considered significant.

## Results

### Effects of fisetin on growth and apoptosis of HSC3 human head and neck cancer cells

To determine the anti-proliferative activity of fisetin on HSC3 cells, cells were treated with fisetin at various concentrations (7.5, 15, and 30 µM) for 24 h and the cell viability was measured using a trypan blue exclusion assay. Fisetin inhibited the viability of HSC3 cells in a concentration-dependent manner Fig. 1B). The association of the apoptosis and the growth inhibitory effects of fisetin was assessed by western blotting with an antibody capable of detecting cleaved PARP. The PARP cleavage was significantly increased in fisetin-treated groups (Fig. 1C). The fisetin-treated HSC3 cells were also stained with the live/dead assay kit or DAPI solution. The dead cells (red stained) were significantly increased after the fisetin treatment (Fig. 1D). In addition, the fisetin-treated HSC3 cells showed prominent nuclear condensation and fragmentation (Fig. 1E). These results indicate that fisetin inhibits cell growth and induces apoptosis in human head and neck cancer cells (HNCCs).

### Involvement of SESN2 in fisetin-induced apoptosis in HSC3 cells

**Fig. 2:**
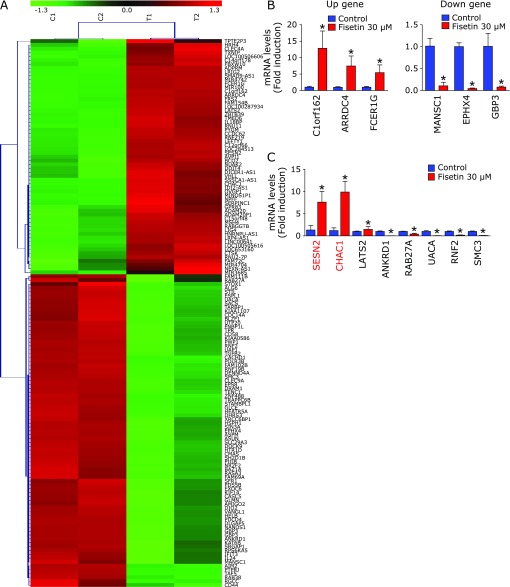
Validation of molecular targets for fisetin-induced apoptosis. (A) Microarray analysis was represented as a heat map showing fold changes (>2) in the expression of regulated genes by fisetin compared with DMSO in HSC3 cells. The color gradient of heat map indicates relative up- or down-regulation than baseline. It represented two independent experiments. From left to right, the first two samples are DMSO-treated groups and the latter is two fisetin-treated samples. Up-regulated genes are shown in red and down-regulated genes are shown in green. mRNA levels of genes regulated by fisetin (B) and candidates related to fisetin-induced apoptosis (C) were measured by real-time PCR. The graphs were expressed as the means of three independent experiments. *p<0.05.

To identify the possible molecular targets for fisetin-induced apoptosis, HSC3 cells were incubated in the presence or absence of 30 µM fisetin and cDNA microarray analysis was performed using Human Gene 2.0 ST Array. The calculated upregulation or downregulation of gene expressions, as determined by the microarray, are listed in Table 2 and 3, respectively. As shown in Table 2 and 3, fisetin-treated HSC3 cells up-regulated 61 genes over 2 folds and down-regulated 81 genes over 2 folds compared with DMSO-treated cells. To verify the reliability, real-time PCR was performed with primers of three different genes, each of which increased (C1orf162, ARRDC4, and FCER1G) or decreased (MANSC1, EPHX4, and GBP3). The results from real-time PCR were similar to those of microarray (Fig. 2B) suggesting that microarray data are reliable. Among 142 genes, 7 apoptosis-associated genes were selected and SESN2 and CHAC1 (ChaC, cation transport regulator homolog 1) changed overwhelmingly compared with other 5 genes (Fig. 2C).

**Fig. 3:**
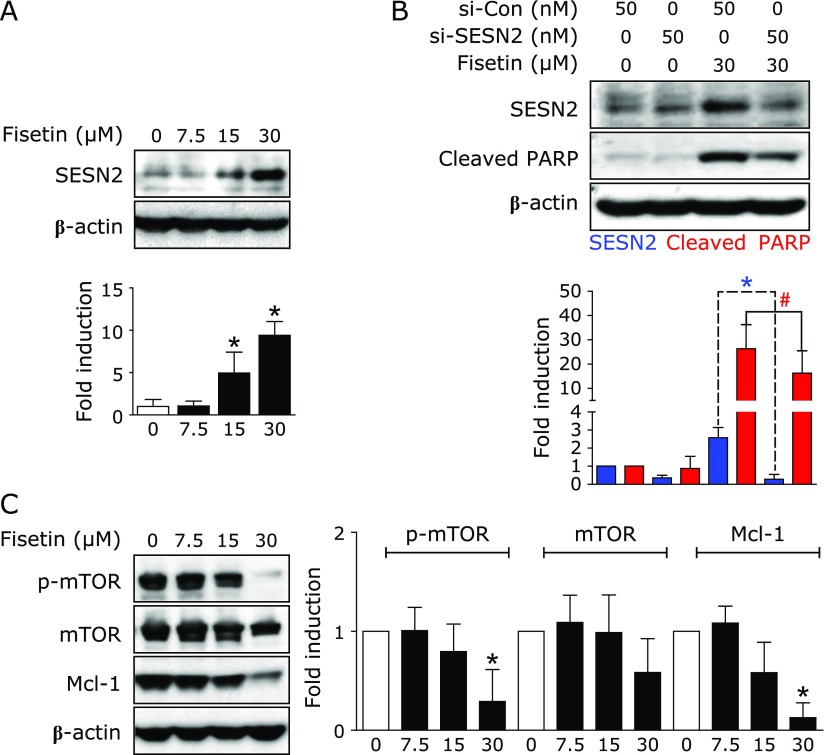
Involvement of SESN2 in fisetin-induced apoptosis. (A) The expression of SESN2 was detected using western blotting. (B) HSC3 cells were transfected with siCon or siSESN2 and then SESN2 and cleaved PARP were examined by western blotting. (C) The expression levels of p-mTOR, mTOR, and Mcl-1 were detected using western blotting. β-Actin was used as a loading control. The all graphs were expressed as the means of three independent experiments. *p<0.05, ^#^*p*<0.05.

Next, we evaluated whether fisetin affects the expression levels of both SESN2 and CHAC1 proteins in HSC3 cells and we observed a significant increase in SESN2 protein expression in HSC3 cells treated with fisetin (Fig. 3A) while CHAC 1 protein was not affected (data not shown). To investigate the functional consequence of the increased SESN2, HSC3 cells were transfected with either siSESN2 or siCon. As shown in Fig. 3B, the knockdown of SESN2 by siSESN2 resulted in significantly less fisetin-induced apoptosis compared to the cells transfected with siCon. These data indicated that SESN2 mediates the apoptosis induced by fisetin in HSC3 cells. Since SESN2 is known to regulate mammalian target of rapamycin (mTOR) and myeloid cell leukemia-1 (Mcl-1) during apoptosis,^(1,17)^ the effects of fisetin on mTOR/Mcl-1 was determined. The results showed that fisetin reduced the protein expression levels of p-mTOR and Mcl-1 (Fig. 3C). Taken together, these findings suggest that fisetin-induced apoptosis may be related to SESN2/mTOR/Mcl-1 signaling axis.

### Growth-inhibitory and apoptotic effects of fisetin via SESN2/mTOR/Mcl-1 in MC3, Ca9.22, and HN22 human head and neck cancer cell lines

**Fig. 4:**
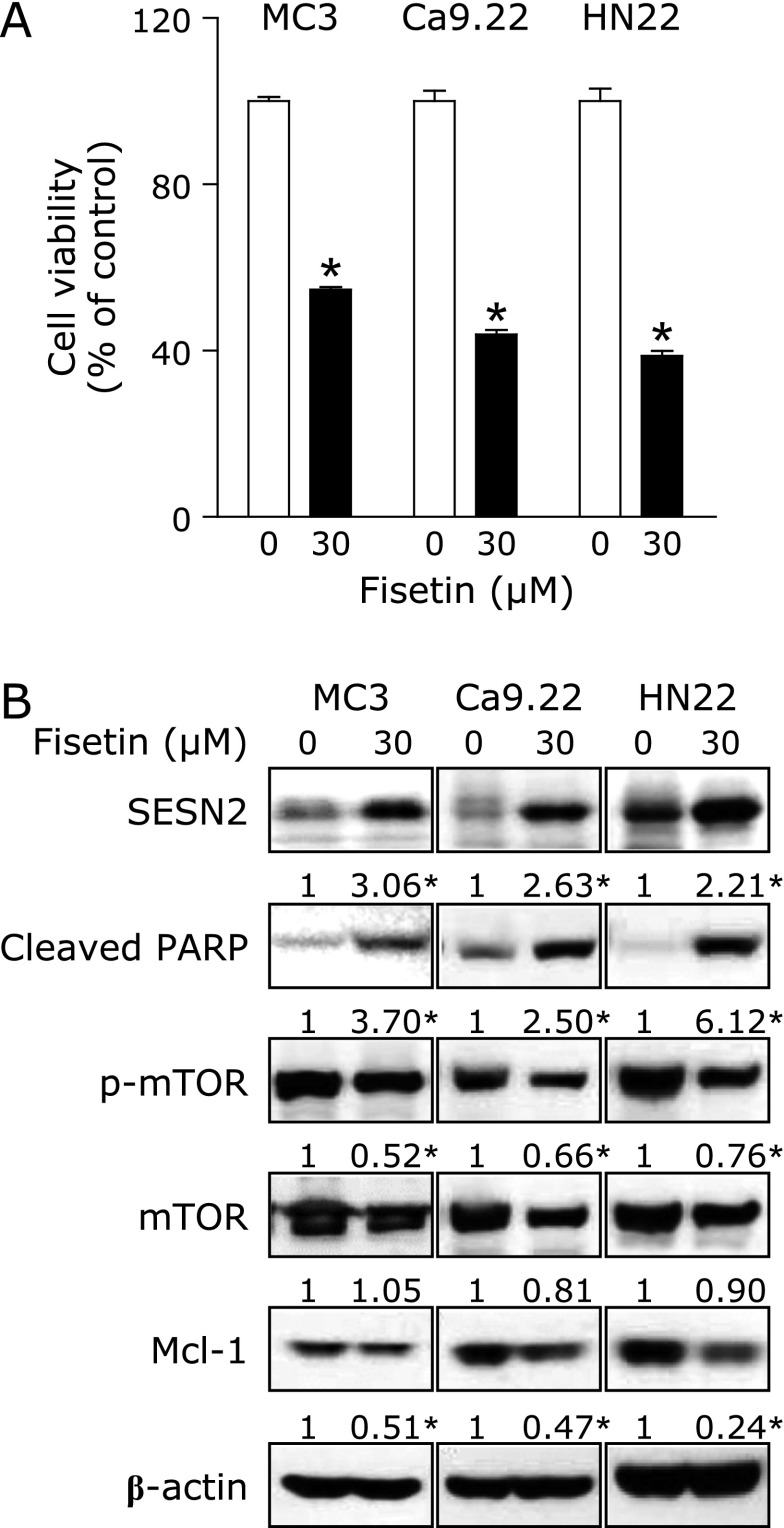
Apoptotic effects of fisetin in human head and neck cancer cell lines. (A) Cell viability was examined using a trypan blue exclusion assay. The graph was expressed as the means of three independent experiments. *p<0.05. (B) SESN2, cleaved PARP, p-mTOR, mTOR, and Mcl-1 were detected using western blotting. β-Actin was used as a loading control.

Three other cell lines (MC3, Ca9.22, and HN22) were used to demonstrate that anti-proliferative and apoptotic efficacy of fisetin was not limited to only HSC3 cell line. The results showed that fisetin treatment significantly suppressed the viability of each cell lines (Fig. 4A) and induced PARP cleavage by inducing a dramatic increase in the expression levels of SESN2 and then reducing the expression levels of p-mTOR and Mcl-1 (Fig. 4B). These findings suggest that fisetin-induced apoptosis via SESN2 in HNCCs is a general mechanism for the anticancer effect of fisetin.

## Discussion

Head and neck cancer (HNCC) is diagnosed in more than 63,000 people in the United States and is responsible for approximately 13,300 death annually in United State.^(18)^ HNCC is a challenging clinical problem and it is recognized that there is a need to develop alternative methods for the management of this tumor. Naturally occurring compounds from plants, vegetables, and fruits have long been used in traditional medicinal systems because of their non-toxic nature in effective dosages.^(20)^ Thus, there is an increasing interest in the efficacy analysis of inhibiting the proliferation of HNCC using phytochemicals with no toxicity. Our research group recently found naturally-derived chemicals such as silymarin, caffeic acid phenethyl ester, and oridonin have anticancer efficacies by inducing apoptosis of HNCCs.^(21–23)^ Fisetin is also naturally derived compound that can be easily synthesized and has no toxicity to normal cells.^(24)^ First, we demonstrated that fisetin reduced cell viability and induced apoptosis in four different HNCCs (HSC3, MC3, Ca9.22, and HN22). There are several evidences to support our present data showing that fisetin suppresses malignant proliferation and induces apoptosis in human oral squamous cell carcinoma and laryngeal carcinoma.^(16,24)^

As mentioned earlier, fisetin has been known to have anticancer efficacies in various types of cancers. It inhibited cell proliferation of cancer cells through modulation of multiple signaling pathways. Fisetin-induced apoptosis of human oral cancer cells was through ROS production and mitochondria-dependent signaling pathways.^(25)^ Fisetin also induced apoptosis through p53-mediated upregulation of death receptor 5 expression in human renal carcinoma cells.^(26)^ However, the molecular targets for fisetin-induced apoptosis in HNCCs are not yet known precisely. Here, using gene expression profiling, we found that fisetin treatment affected 142 genes (61 up-regulated genes and 81 down-regulated genes). Among them, the mRNA expression levels of SESN2 and CHAC1, which are apoptosis-associated genes, were significantly up-regulated in fisetin-treated HSC3 cells. Fisetin also increased the expression level of SESN2 protein and knockdown of SESN2 by a siRNA technique clearly recovered HSC3 cells from fisetin-induced apoptosis, suggesting that SESN2 is involved in fisetin-mediated apoptosis. Consistent with our data, it was reported that quercetin, which flavonoid and structurally similar to fisetin, regulates the SENS2 signaling and induces apoptosis by inducing the generation of intracellular ROS and AMPK/p38 signaling pathway.^(27)^ It suggests the possibility of SESN2 as a therapeutic target for cancer treatment. To our best knowledge, this is the first report demonstrating that SESN2 may be a molecular target for fisetin-induced apoptosis in HNCCs. CHAC1 was identified as a component of the unfolded protein response (UPR) signaling pathway. Fisetin induced apoptosis through endoplasmic reticulum (ER) stress in oral cancer and non-small cell lung cancer.^(25,28,29)^ However, we found that fisetin treatment did not alter the expression level of CHAC1 protein while it significantly augmented its mRNA level in the present study. These data indicate that fisetin-induced apoptosis may not be associated with ER stress in HSC3 HNCCs.

The mammalian target of rapamycin (mTOR) is a serine-threonine protein kinase that are resistant to the growth-inhibitory activities of rapamycin. mTOR comprises of two distinct multiprotein complexes; mTORC1 and mTORC2. SESN2 inhibits mTOR-dependent phosphorylation of p70S6K and 4E-BP1,^(6)^ and knockdown of SESN2 resulted in the activation of mTOR signaling indicating that the important role of SESN2 on mTOR inhibition.^(30)^ Our results from western blotting demonstrated that mTOR was down-regulated by fisetin treatment in HNCCs consistent with other previous studies showing tumor inhibiting potentials of fisetin through mTOR pathway.^(24,31,32)^ These results suggest that fisetin-induced apoptotic potentials is through SESN2/mTOR signaling axis.

In conclusion, fisetin suppresses the growth of HNCCs and induces apoptotic cell death. Its functional role is attributed to SESN2/mTOR/Mcl-1 signaling axis. On the basis of our findings, we suggest that targeting SESN2 by fisetin or its derivatives could be a new strategy to overcome HNCCs.

## Author Contributions

DH Won and SH Chung performed experiments and drafted the manuscript; JA Shin, KO Hong, IH Yang were responsible for the study design and data analysis; JW Yun and SD Cho designed the study and revised the manuscript. All authors reviewed and approved the final manuscript.

## Figures and Tables

**Table 1:** Primer sequences used for real-time PCR

**Gene**		**Sequences (5**'**→3**'**)**
C1orf162	Forward:	ATCCTCCAGCCAAGCTTTC
	Reverse:	ATGGTTCTCAGCCAAGTGATT

ARRDC4	Forward:	CACATTCCTCCTTACCCTCAAC
	Reverse:	GATGTGGGTCAACCTCTGAATAA

FCER1G	Forward:	GATGTGGGTCAACCTCTGAATAA
	Reverse:	CCGTGTAAACACCATCTGATTTC

MANSC1	Forward:	AGGCAGCTTAGAAACCATACC
	Reverse:	GGAAGACTC CACATTTGACATAGA

EPHX4	Forward:	TTCTCAGCCTGGAGCATTAAG
	Reverse:	CCACAGTAGTAGTGTTGGAGTG

GBP3	Forward:	AGAGCCTAGTGCTGACCTATATC
	Reverse:	TGCGGCTGAGTTCTCTATCT

SESN2	Forward:	GCGGAACCTCAAGGTCTATATC
	Reverse:	AAGTTCACGTGGACCTTCTC

CHAC1	Forward:	GTGCTTGGTGGCTACGATAC
	Reverse:	CACATAGGCCAATGCCTTCA

LATS2	Forward:	GTAGATGAAGAAAGCCCTTGGA
	Reverse:	GTGCTCAGGATGCTTGTTATTG

ANKRD1	Forward:	GGTGAGACTGAACCGCTATAAG
	Reverse:	GGTTCCATTCTGCCAGTGTA

RAB27A	Forward:	AGGACCAGAGAGTAGTGAAAGA
	Reverse:	CGGCTTATGTTTGTCCCATTG

UACA	Forward:	CCTTTCCCAACTCACCTACAC
	Reverse:	CAGCTGTTGCTCCAGAGATT

RNF2	Forward:	CAAGTATCTGGCTGTGAGGTTAG
	Reverse:	CTGCTTCTCACTGGCTGTATC

SMC3	Forward:	GGTGGACAGAAATCCTTGGTAG
	Reverse:	ATCCAGAGCCTGGTCAATTTC

GAPDH	Forward:	GTGGTCTCCTCTGACTTCAAC
	Reverse:	CCTGTTGCTGTAGCCAAATTC

**Table 2:** Upregulation of gene expressions in fisetin-treated HSC3 cells

**Gene symbol**	**Gene bank no.**	**Gene description**	**Relative expression**
*MIR100*	NR_029515	microRNA 100	18.6201
*SMAD9-AS1*	ENST00000437983	*SMAD9 antisense RNA 1*	10.0527
*MIR4742*	NR_039896	*microRNA 4742*	7.0163
*C1orf162*	ENST00000343534	*chromosome 1 open reading frame 162*	5.1798
*ARRDC4*	ENST00000268042	*arrestin domain containing 4*	5.1262
*FCER1G*	NM_004106	*Fc fragment of IgE, high affinity I, receptor for; gamma polypeptide*	5.0681
*SESN2*	NM_031459	*sestrin 2*	4.7266
*C14orf178*	ENST00000439131	*chromosome14 open reading frame 178*	4.2350
*LOC100506606*	AK090590	*uncharacterized LOC100506606*	4.1875
*LRP4-AS1*	NR_038909	*LRP4 antisense RNA 1*	4.1462
*ADAM20P1*	NR_037933	*ADAM etallopeptidasedomain 20 pseudogene 1*	4.1350
*IDI2-AS1*	NR_027709	*IDI2 antisense RNA 1*	4.1319
*LINC00641*	NR_038971	*long intergenic non-protein coding RNA 641*	4.0861
*CCDC62*	ENST00000253079	*coiled-coil domain containing 62*	3.8391
*HNRNPU-AS1*	NR_026778	*HNRNPU antisense RNA 1*	3.8236
*NUAK2*	ENST00000367157	*NUAK family, SNF1-like kinase, 2*	3.7584
*PYGM*	NM_005609	*phosphorylase, glycogen, muscle*	3.7578
*FBXW10*	NR_051988	*F-box and WD repeat domain containing 10*	3.4988
*HRH4*	NM_021624	*histamine receptor H4*	3.4564
*MIR3685*	NR_037456	*microRNA 3685*	3.4463
*GPR83*	NM_016540	*G protein-coupled receptor 83*	3.3115
*TMED6*	NM_144676	*transmembrane emp24 protein transport domain containing 6*	3.2819
*DDIT4*	NM_019058	*DNA-damage-inducible transcript 4*	3.2205
*NPFF*	NM_003717	*neuropeptide FF-amide peptide precursor*	3.1103
*RNU11*	NR_004407	*RNA, U11 small nuclear*	3.0542
*SERPINC1*	NM_000488	*serpin peptidase inhibitor, clade C (antithrombin), member 1*	2.9955
*RNU2-7P*	ENST00000410794	*RNA, U2 small nuclear 7, pseudogene*	2.9560
*CHAC1*	ENST00000446533	*ChaC, cation transport regulator homolog 1 (E. coli)*	2.9385
*SSSCA1-AS1*	NR_038923	*SSSCA1 antisense RNA 1 (head to head)*	2.7727
*ADAM20*	NM_003814	*ADAM metallopeptidase domain 20*	2.7683
*ADPRM*	NM_020233	*ADP-ribose/CDP-alcohol diphosphatase, manganese-dependent*	2.7642
*CLEC4A*	NM_016184	*C-type lectin domain family 4, member A*	2.7375
*FAM72C*	ENST00000492131	*family with sequence similarity 72, member C*	2.6754
*DICER1-AS1*	NR_015415	*DICER1 antisense RNA 1*	2.6597
*MINOS1P1*	NR_051980	*mitochondrial inner membrane organizing system 1 pseudogene 1*	2.6291
*LOC100287934*	AK290103	*uncharacterized LOC100287934*	2.6185
*C12orf66*	ENST00000398055	*chromosome 12 open reading frame 66*	2.5818
*RNF219*	NM_024546	*ring finger protein 219*	2.5665
*LRIG2*	NM_014813	*leucine-rich repeats and immunoglobulin-like domains 2*	2.5337
*TXNIP*	NM_006472	*thioredoxin interacting protein*	2.5048
*LEFTY1*	NM_020997	*left-right determination factor 1*	2.4855
*MIR4704*	NR_039853	*microRNA 4704*	2.4683
*MSH4*	NM_002440	*mutS homolog 4 (E. coli)*	2.4679
*LOC284513*	AK096098	*uncharacterized LOC284513*	2.4190
*BCO2*	NM_031938	*beta-carotene oxygenase 2*	2.4055
*RABGGTB*	NR_073562	*Rab eranylgeranyltransferase, beta subunit*	2.3985
*NEXN-AS1*	ENST00000421331	*NEXN antisense RNA 1*	2.3835
*OVGP1*	NM_002557	*oviductal glycoprotein 1, 120kDa*	2.3758
*AVPI1*	ENST00000370626	*arginine vasopressin-induced 1*	2.3462
*C15orf48*	NM_032413	*chromosome 15 open reading frame 48*	2.2732
*LOC100505616*	AL360137	*uncharacterized LOC100505616*	2.2586
*FAM154B*	AK304339	*family with sequence similarity 154, member B*	2.2529
*VHLL*	NM_001004319	*von Hippel-Lindau tumor suppressor-like*	2.2319
*CTSK*	NM_000396	*cathepsin K*	2.2017
*FRS2*	NM_006654	*fibroblast growth factor receptor substrate 2*	2.1753
*LOC653160*	NR_037869	*uncharacterized LOC653160*	2.1697
*ZBTB39*	NM_014830	*zinc finger and BTB domain containing 39*	2.1601
*LIG4*	NM_002312	*ligase IV, DNA, ATP-dependent*	2.1438
*LATS2*	NM_014572	*large tumor suppressor kinase 2*	2.1389
*TPTE2P3*	NR_002793	*transmembrane phosphoinositide 3-phosphatase and tensin homolog 2 pseudogene 3*	2.1301
*IL18BP*	NM_005699	*interleukin 18 binding protein*	2.0777

**Table 3:** Downregulation of gene expressions in fisetin-treated HSC3 cells

**Gene symbol**	**Gene bank no.**	**Gene description**	**Relative expression**
*ANKRD1*	NM_014391	*ankyrin repeat domain 1 (cardiac muscle)*	–5.7362
*RAB27A*	ENST00000563262	*RAB27A, member RAS oncogene family*	–3.7658
*GBP3*	ENST00000564037	*guanylate binding protein 3*	–3.6909
*MANSC1*	NM_018050	*MANSC domain containing 1*	–3.6613
*SLC29A3*	NM_018344	*solute carrier family 29 (nucleoside transporters), member 3*	–3.5454
*EPHX4*	NM_173567	*epoxide hydrolase 4*	–3.4609
*SFR1*	NM_145247	*SWI5-dependent recombination repair 1*	–3.2982
*IL24*	NM_006850	*interleukin 24*	–3.2138
*CASC5*	NM_170589	*cancer susceptibility candidate 5*	–3.1766
*DLGAP5*	NM_014750	*discs, large (Drosophila) homolog-associated protein 5*	–3.1121
*HSPH1*	NM_006644	*heat shock 105kDa/110kDa protein 1*	–3.0430
*FAM111B*	NM_198947	*family with sequence similarity 111, member B*	–3.0397
*PDCD4*	NM_145341	*programmed cell death 4 (neoplastic transformation inhibitor)*	–3.0233
*SRGAP1*	ENST00000355086	*SLIT-ROBO Rho GTPase activating protein 1*	–3.0089
*ASUN*	NM_018164	*asunder spermatogenesis regulator*	–2.9678
*BAZ1A*	NM_013448	*bromodomain adjacent to zinc finger domain, 1A*	–2.9335
*GBP3*	NM_018284	*guanylate binding protein 3*	–2.9132
*UACA*	NM_018003	*uveal autoantigen with coiled-coil domains and ankyrin repeats*	–2.9130
*ASPM*	NM_018136	*asp (abnormal spindle) homolog, microcephaly associated (Drosophila)*	–2.8024
*FAM69A*	NM_001252271	*family with sequence similarity 69, member A*	–2.7892
*TAF5*	NM_006951	*TAF5 RNA polymerase II, TATA box binding protein (TBP)-associated factor, 100kDa*	–2.7883
*HEATR5A*	NM_015473	*HEAT repeat containing 5A*	–2.7877
*KIF14*	NM_014875	*kinesin family member 14*	–2.7846
*POLR3B*	NM_018082	*polymerase (RNA) III (DNA directed) polypeptide B*	–2.7774
*SH2D1B*	NM_053282	*SH2 domain containing 1B*	–2.7574
*NANOS1*	NM_199461	*nanos homolog 1 (Drosophila)*	–2.7510
*XRCC6BP1*	ENST00000300145	*XRCC6 binding protein 1*	–2.7489
*GLCE*	NM_015554	*glucuronic acid epimerase*	–2.7447
*TGFB2*	NM_001135599	*transforming growth factor, beta 2*	–2.7301
*ZNF488*	NM_153034	*zinc finger protein 488*	–2.7141
*CD58*	NM_001779	*CD58 molecule*	–2.7008
*HELB*	ENST00000247815	*helicase (DNA) B*	–2.6975
*GLMN*	NM_053274	*glomulin, FKBP associated protein*	–2.6815
*STAMBPL1*	NM_020799	*STAM binding protein-like 1*	–2.6728
*CDC14A*	NM_003672	*cell division cycle 14A*	–2.6448
*KIAA1107*	NM_015237	*KIAA1107*	–2.6375
*IFIT3*	NM_001031683	*interferon-induced protein with tetratricopeptide repeats 3*	–2.6349
*EXOC6*	NM_001013848	*exocyst complex component 6*	–2.6189
*FAM102B*	ENST00000370035	*family with sequence similarity 102, member B*	–2.6185
*RNF2*	NM_007212	*ring finger protein 2*	–2.6137
*FNBP1L*	NM_001024948	*formin binding protein 1-like*	–2.5804
*PIGB*	NM_004855	*phosphatidylinositol glycan anchor biosynthesis, class B*	–2.5770
*RPS6KA5*	NM_004755	*ribosomal protein S6 kinase, 90kDa, polypeptide 5*	–2.5702
*RASA3*	NM_007368	*RAS p21 protein activator 3*	–2.5530
*KAT6B*	NM_012330	*K(lysine) acetyltransferase 6B*	–2.5422
*VANGL1*	NM_001172412	*VANGL planar cell polarity protein 1*	–2.5304
*PWP1*	NM_007062	*PWP1 homolog (S. cerevisiae)*	–2.4969
*TPR*	NM_003292	*translocated promoter region, nuclear basket protein*	–2.4862
*CACHD1*	NM_020925	*cache domain containing 1*	–2.4845
*HTR1D*	NM_000864	*5-hydroxytryptamine (serotonin) receptor 1D, G protein-coupled*	–2.4792
*CMAS*	NM_018686	*cytidine monophosphate -acetylneuraminic acid synthetase*	–2.4709
*UAP1*	NM_003115	*UDP-N-acteylglucosamine pyrophosphorylase 1*	–2.4536
*PDS5B*	NM_015032	*PDS5, regulator of cohesion maintenance, homolog B (S. cerevisiae)*	–2.4324
*NR2F2*	NM_001145155	*nuclear receptor subfamily 2, group F, member 2*	–2.4221
*KIAA0586*	NM_001244189	*KIAA0586*	–2.4081
*RGCC*	NM_014059	*regulator of cell cycle*	–2.3926
*TENC1*	ENST00000314250	*tensin like C1 domain containing phosphatase (tensin 2)*	–2.3821
*AMIGO2*	NM_001143668	*adhesion molecule with Ig-like domain 2*	–2.3782
*SIN3A*	NM_001145358	*SIN3 transcription regulator homolog A (yeast)*	–2.3752
*DIO2*	NM_001242502	*deiodinase, iodothyronine, type II*	–2.3311
*STOX1*	NM_152709	*storkhead box 1*	–2.3298
*DOCK9*	NM_001130048	*dedicator of cytokinesis 9*	–2.3213
*DHRS3*	NM_004753	*dehydrogenase/reductase (SDR family) member 3*	–2.3195
*UTP20*	NM_014503	*UTP20, small subunit (SSU) processome component, homolog (yeast)*	–2.3095
*SMC3*	NM_005445	*structural maintenance of chromosomes 3*	–2.2974
*AIM2*	NM_004833	*absent in melanoma 2*	–2.2717
*DENND4A*	NM_001144823	*DENN/MADD domain containing 4A*	–2.2705
*CLEC9A*	NM_207345	*C-type lectin domain family 9, member A*	–2.2468
*DRAM1*	NM_018370	*DNA-damage regulated autophagy modulator 1*	–2.2426
*RNF19B*	NM_153341	*ring finger protein 19B*	–2.2414
*EPS8*	NM_004447	*epidermal growth factor receptor pathway substrate 8*	–2.2327
*SACS*	NM_014363	*spastic ataxia of Charlevoix-Saguenay (sacsin)*	–2.2269
*TARBP1*	NM_005646	*TAR (HIV-1) RNA binding protein 1*	–2.1744
*RAB38*	NM_022337	*RAB38, member RAS oncogene family*	–2.1648
*CD44*	AF086543	*CD44 molecule (Indian blood group)*	–2.1532
*STIL*	NM_001048166	*SCL/TAL1 interrupting locus*	–2.1308
*TRAPPC6B*	NM_001079537	*trafficking protein particle complex 6B*	–2.1090
*ALG6*	NM_013339	*ALG6, alpha-1,3-glucosyltransferase*	–2.0650
*ESPL1*	NM_012291	*extra spindle pole bodies homolog 1 (S. cerevisiae)*	–2.0576
*BLZF1*	NM_003666	*basic leucine zipper nuclear factor 1*	–2.0247
*PTPRJ*	NM_002843	*protein tyrosine phosphatase, receptor type, J*	–2.0105
